# Global-scale river flood vulnerability in the last 50 years

**DOI:** 10.1038/srep36021

**Published:** 2016-10-26

**Authors:** Masahiro Tanoue, Yukiko Hirabayashi, Hiroaki Ikeuchi

**Affiliations:** 1 Institute of Engineering Innovation, The University of Tokyo, 2-11-16 Yayoi, Bunkyo-ku, Tokyo 113-8656, Japan; 2 Department of Civil Engineering, The University of Tokyo, 7-3-1 Hongo, Bunkyo-ku, Tokyo 113-8656, Japan

## Abstract

The impacts of flooding are expected to rise due to population increases, economic growth and climate change. Hence, understanding the physical and spatiotemporal characteristics of risk drivers (hazard, exposure and vulnerability) is required to develop effective flood mitigation measures. Here, the long-term trend in flood vulnerability was analysed globally, calculated from the ratio of the reported flood loss or damage to the modelled flood exposure using a global river and inundation model. A previous study showed decreasing global flood vulnerability over a shorter period using different disaster data. The long-term analysis demonstrated for the first time that flood vulnerability to economic losses in upper-middle, lower-middle and low-income countries shows an inverted U-shape, as a result of the balance between economic growth and various historical socioeconomic efforts to reduce damage, leading to non-significant upward or downward trends. We also show that the flood-exposed population is affected by historical changes in population distribution, with changes in flood vulnerability of up to 48.9%. Both increasing and decreasing trends in flood vulnerability were observed in different countries, implying that population growth scenarios considering spatial distribution changes could affect flood risk projections.

River floods are one of the most common natural hazards, causing devastating impacts worldwide. Previous studies have indicated that increased exposure of people and assets, as a result of population increase and economic growth, has caused more damage due to weather-related natural disasters including flooding^1^,^2^. In addition, climate change may increase the frequency or magnitude of flooding^3^,^4^,^5^. The impact of flooding is especially harmful in developing countries due to low levels of flood protection. For example, 6,648 flood fatalities were recorded in 2013 in India and Nepal, while the Philippines has suffered from recurring flooding that caused more than 100 fatalities every year between 2011 and 2013, and prolonged flooding in Thailand in 2011 caused serious economic losses^6^. Developed countries have also suffered from flooding: in Europe, the Danube flooded in 2013, as did the Kinu River in Japan in 2015. However, flood loss and damage, especially in terms of numbers of fatalities, are generally less severe in developed versus developing countries due to historical efforts to mitigate flood impacts^7^. Differences in flood risk among regions reflect the balance between the magnitude of the flood (hazard), the number of people or the value of assets potentially affected by flooding (exposure), and the susceptibility to harm or lack of socioeconomic capacity to cope with flood risk depending on economic, social, demographic, cultural, institutional and governance factors (vulnerability)^2^,^8^,^9^,^10^,^11^,^12^. As these controlling risk factors change geographically and temporally, an understanding of their physical and spatiotemporal characteristics is essential for the projection of future flood risks and the development of effective flood mitigation measures.

Several studies have assessed the spatiotemporal characteristics of flood hazard and exposure at regional and global scales under current and future climate conditions^3^,^5^,^13^,^14^,^15^,^16^,^17^,^18^. For example, Ward *et al*.^16^ presented spatial maps of modelled flood risk (affected population, affected gross domestic product [GDP], affected agricultural value and urban damage) according to flood hazard level (i.e., return period) using a global river and inundation model. Hirabayashi *et al*.^3^ projected the increase in the population potentially exposed to flooding under climate change conditions obtained from several climate models. Winsemius *et al*.^5^ separated the damage of climate change and socio-economic development on flood risk projection. These studies calculated exposure in terms of the number of people or the value of assets potentially affected by flooding under different flood hazard levels (e.g., magnitude of modelled discharge). Most of these studies did not consider spatiotemporal changes in flood vulnerability (i.e., flood protection levels) due to a lack of data for this parameter, while Winsemius *et al*.^5^ demonstrated that the improvement of flood protection level significantly reduced the damage. Other studies have attempted to estimate historical spatiotemporal patterns of vulnerability using a quantification model at a national scale^19^,^20^,^21^; however, these methodologies are applicable only to local scales due to limited data availability^22^.

One recent study^10^ (hereafter referred to as J15) proposed a method to calculate flood vulnerability at the global scale in terms of the ratio of the reported flood loss and damage to the modelled flood exposure, obtained by using a global river and inundation model without considering any flood mitigation measures. Using this concept, historical improvements in flood vulnerability between 1990 and 2010 were successfully presented. The method presented in J15 for estimation of flood vulnerability is novel and highly useful; however, it would be beneficial to confirm these findings using an independent global river and inundation model and different disaster statistics, because large uncertainties in land surface and hydrological modelling^23^,^24^ and inconsistencies among disaster statistics^25^ have been demonstrated previously. In addition, because dramatic improvements in flood protection levels occurred before 1990 in some developed countries (e.g. in Japan), analysis of longer periods, i.e., including years before 1990, should provide additional information on spatiotemporal changes in flood vulnerability.

Therefore, in this paper, we show long-term trends in flood vulnerability worldwide between 1960 and 2013. Historical flood exposures, in terms of populations and assets potentially affected by flooding (calculated by a state-of-the-art global river and inundation model)^26^ – and reported flood disaster statistics from the Emergency Events Database (EM-DAT)^6^ – were used to calculate flood vulnerability. We calculated annual modelled flood exposure from 1960 to 2013 by overlaying global gridded population data from the History Database of the Global Environment (HYDE, ver. 3.1)^27^,^28^ or the national nominal GDP per capita of the World Bank multiplied by the gridded population data onto the modelled annual maximum flooded area fraction. See “Modelled flood exposure calculation” in Methods for details. Following J15, we define flood vulnerability in terms of the mortality rate (the ratio of the reported fatalities to the modelled exposed population) and the loss rate (the ratio of the reported economic losses to the modelled exposed GDP). See Methods “Flood vulnerability calculation” for details. We then analyse the effect of historical changes in the spatial distribution of populations on flood exposure and vulnerability, because, even though several studies have identified both increasing flood exposures due to unplanned human settlements in flood-prone regions^29^,^30^ and decreasing flood exposures as a result of autonomous adaptations (such as land use regulation and displacement of people to safer regions^31^), no study has yet quantified the temporal distribution or magnitude of these effects.

## Results

### Global flood impact and exposure

**Figure 1:**
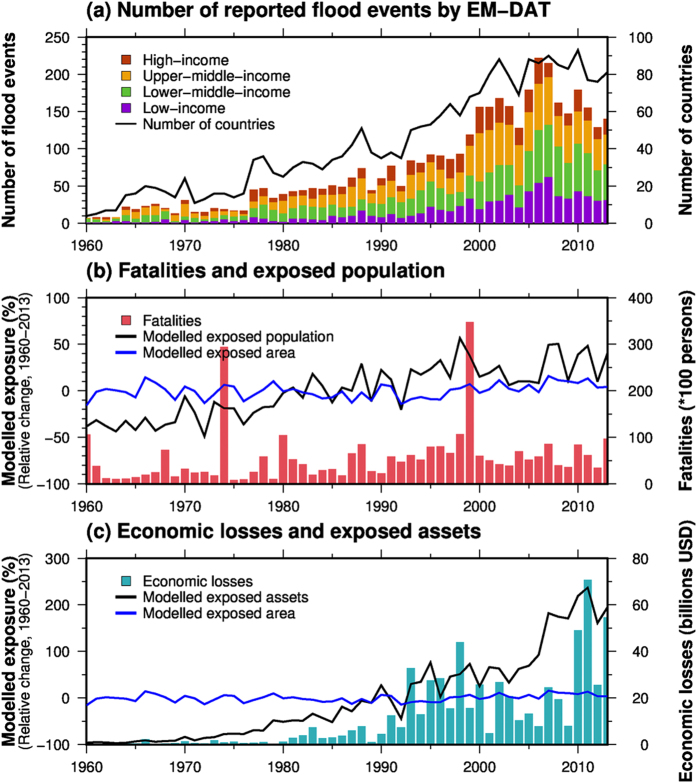
(**a**) Number of flood events for each income level (bar) and number of countries in which a flood event was reported in the Emergency Events Database (EM-DAT) (solid line) from 1960 to 2013. The definition of income level was obtained from the World Bank (http://data.worldbank.org/about/country-and-lending-groups). (**b**) Reported fatalities (bar), the modelled global exposed population (black line) and modelled global flooded area (blue line). The modelled global exposed population and flooded area show relative changes compared to the average across 1960–2013. (**c**) Same as (**b**) but for reported economic loss according to flooding and modelled global exposed GDP. This figure was created using Generic Mapping Tools 4.5.6.

The number of flood events reported in the EM-DAT disaster database has increased since the 1960s (Fig. 1a). Flood events have been recorded in the EM-DAT if the event fulfilled the criteria (see “Flood vulnerability calculation” in Methods). However, many events that meet the criteria are often not recorded in the EM-DAT. For example, the number of countries in which flood events were reported in the EM-DAT was less than 50 until the mid-1980 s, reaching around 80 after 2000. In particular, the number of flood events reported in low-income countries was very low before the mid-1990 s. This increasing trend in flood events is not due to an increasing number of flood hazards worldwide, as the total global modelled flooded areas without consideration of flood mitigation measures show a much lower increasing trend (0.15% per year from 1960 to 2013, blue line in Fig. 1b,c); this indicates that one of the main drivers of the increased number of flood reports is improvements in data accessibility and information gathering. Other factors (socioeconomic changes such as population increase) could also contribute.

The annual total number of reported fatalities and the value of reported economic losses from flooding in the EM-DAT show an increasing trend between 1960 and 2013 (1.5% per year for the annual total number of reported fatalities and 6.3% per year for the value of reported economic losses; bars in Fig. 1b,c). Because the global modelled exposed area does not show a significant increase, the number of flood events reported in the EM-DAT and the upward trend in flood exposure may reflect an increase in socioeconomic development (1.5% per year for the modelled exposed population and 5.6% per year for the modelled exposed GDP; black lines in Fig. 1b,c), manifested for example in increases in population density or assets in flood-prone regions.

**Figure 2:**
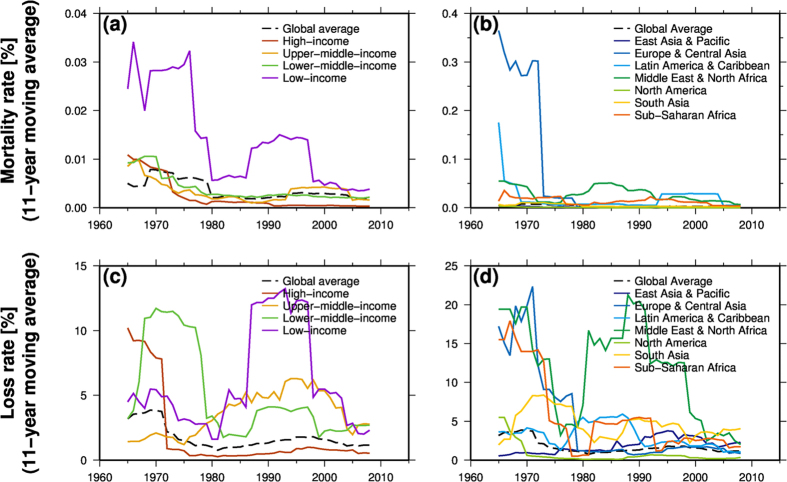
Mortality rate (**a,b**) and loss rate (**c,d**) at each income level (**a,c**) and each region (**b,d**). An 11-year moving average is shown. The present-day (base year 2014) income level for each country and regional division were derived from the World Bank. This figure was created using Generic Mapping Tools 4.5.6.

To check whether our model reasonably replicates past flooding, the summed data, on the modelled exposed populations and exposed GDP of all countries, were calculated (see the “Flood Exposure Calculation” section for details) and compared with reported fatalities and economic losses in the EM-DAT. The increasing trend in reported fatalities and economic losses in the EM-DAT was also seen in the modelled exposed population and the modelled exposed GDP, respectively (Fig. 2b,c). However, the modelled exposed population data show a weak correlation with the reported fatalities (R = 0.33). When two devastating flood events that caused an enormous number of reported fatalities (28,700 fatalities in Bangladesh in 1974 [98% of global flood-related fatalities for that year] and 30,005 fatalities in Venezuela in 1999 [86% of global flood-related fatalities for that year]) were excluded, the correlation coefficient improved (R = 0.52). Country level analysis for Bangladesh between 1980–2013 showed that the modelled exposed population could capture historical large flood events with large numbers of reported fatalities fairly well, leading to a stronger correlation (R = 0.55) between the modelled exposed population and the reported fatalities (see details in Supplementary Information and Supplementary Fig. S4). A similar analysis for 30 countries, where flood events were recorded in the EM-DAT in at least 20 years between 1980 and 2013, showed that 25 countries displayed positive correlation between the reported fatalities and the modelled exposed population (Supplementary Fig. S5a), although low correlations were observed in countries with low flood vulnerability or that rarely suffered from flooding. Modelled exposed GDP shows a good correlation with reported economic losses (R = 0.80), maybe due to a rapid increase in assets in flood-prone regions associated with economic growth, which can be easily replicated when observed GDP data are used.

### Spatiotemporal characteristics of flood vulnerability

**Figure 3:**
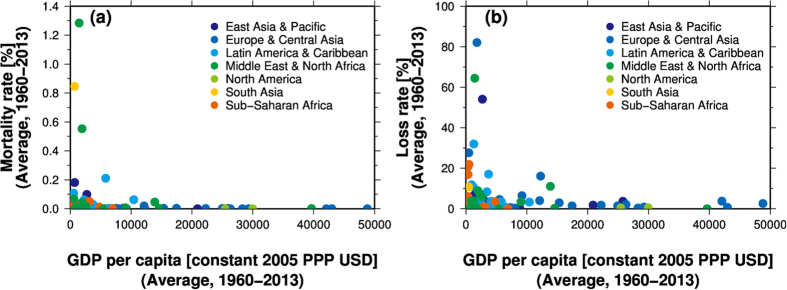
(**a**) Relationship between gross domestic product (GDP) per capita (USD at purchasing power parity [PPP] in 2005) and mortality rate. (**b**) Same as (**a**) but for loss rate. GDP per capita was derived from James *et al*.^41^. The present-day regional divisions were derived from the World Bank. This figure was created using Generic Mapping Tools 4.5.6.

The 11-year moving average of global mortality rate (the ratio of reported fatalities to the modelled exposed population) shows a significant (P < 0.01) negative trend between 1960 and 2013 (Fig. 2a). Mortality rates at all income levels also show negative trends over the same period. At the regional scale, a negative trend in mortality rate is seen in East Asia and the Pacific, Europe and Central Asia, Latin America and the Caribbean, and the Middle East and North Africa. The mortality rate is higher in countries with a lower income level (Fig. 2b). Country level analysis of GDP per capita and mortality rates confirms that the mortality rate decreases when GDP per capita increases (Fig. 3a). The mortality rate is greater than 0.1% (1 fatality per thousand modelled exposed population) only when GDP per capita is less than 10,000 USD, and the mortality rate does not exceed 0.1% in high-income countries. In addition, high mortality rates (>0.5%) are seen in countries in dry inland regions including the Middle East and Africa.

The global loss rate (the ratio of the reported economic losses to the modelled exposed GDP) also shows a significant (P < 0.05) negative trend in the period 1960–2013 (Fig. 2c). Although an inverse relation is also found between loss rate and GDP per capita (Fig. 3b), the loss rate varies between different income levels (Fig. 2c). The loss rate in countries with lower-middle income shows a negative trend, while that in countries with upper-middle income shows a positive trend.

The loss rates in high-income countries dropped in the middle of the 1970 s and remained low in subsequent years (Fig. 2a). On the other hand, all other countries showed an inverted U-shape pattern. For example, the loss rates in upper-middle-income countries showed an increase in loss rate in the period from 1975 to 2000 and a decrease after 2000. The regional average loss rates show a negative trend in all regions except for East Asia and the Pacific (Fig. 2d). The loss rates were higher than 10% (100,000 USD economic losses per million modelled exposed GDP) in countries where the GDP per capita was less than 15,000 USD.

### Effect of change in population distribution on flood exposure and vulnerability calculation

The negative trend in calculated flood vulnerability (mortality and loss rates) reflects all of the historical man-made efforts to reduce flood risk. As a result, flood vulnerability has changed over time and space depending on local socioeconomic development conditions, including flood protection measures (e.g., levees, dams, river management, dredging, flood warning systems and land use management), topography and hydro-climatic conditions. At the same time, flood vulnerability is greatly affected by spatiotemporal changes in populations and assets. For example, several studies indicate that unplanned urbanisation and human settlements in flood-prone regions have increased flood risk in Africa^30^ and low coastal regions^29^, whereas displacement of people to safer areas from flood-prone regions is expected to reduce flood risk^31^. We therefore investigated how flood exposure changes in response to spatiotemporal changes in the population and showed its potential effect on the flood vulnerability calculation.

**Figure 4:**
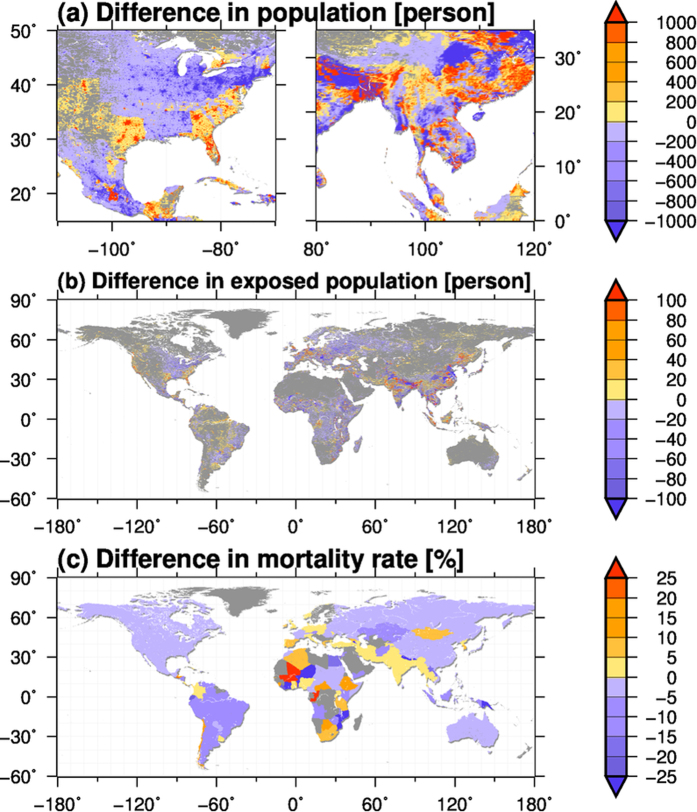
(**a**) Difference between the population with a fixed distribution in 1960 (FPOP) and the real population (RPOP) in the North American continent (left) and south-eastern part of Eurasian continent (right). The average for the period 1991–2000 is shown. (**b**) Same as (**a**), but for the modelled exposed population. (**c**) Difference between the mortality rates calculated using FPOP and RPOP. Positive (negative) values indicate an increase (decrease) in mortality rate due to the change in population distribution within a country since 1960. This figure was created using Generic Mapping Tools 4.5.6.

Figure 4a compares the mean difference between the real population map (hereafter RPOP) and a population distribution identical to that in 1960, but scaled to obtain the real population of a country for each year (hereafter FPOP). Because the country population is identical in both datasets, the difference between the datasets reflects changes within the population distribution of a country since 1960. Population increases in urban regions, and decreases in rural regions, were observed in many countries including China, Thailand and the USA, reflecting demographic changes associated with urban built-up areas and land use changes.

The differences in population distribution affect the modelled exposed population, and hence the mortality rate (Fig. 4b,c). The differences in the modelled exposed population were large in Asia and Africa, resulting in large changes in the mortality rate (up to 48.9%) in these countries. Reductions in the modelled exposed population and improvements in mortality rate due to changing population distributions were found in 58 out of 113 analysed countries, indicating that people have tended to live in less flood-vulnerable regions in these countries since 1960. The reduction in mortality rate was large (>20%) in Armenia, Benin, Bhutan, Côte d’Ivoire, Ecuador, Jamaica, Laos, Liberia, Mozambique, Nepal, Niger and Papua New Guinea. In contrast, changes in the population distribution led to an increase in the modelled exposed population, and a worse mortality rate, in 55 countries. Large increases in mortality rate (>20%) were observed in Botswana, Burkina Faso, Chad, Chile, El Salvador, Ethiopia, Israel, Mali, South Korea and Taiwan.

## Discussion

The improvements in mortality rates and in loss rates partly reflect various historical socioeconomic efforts to reduce flood risk (Fig. 3). Low-income countries experience significant damage from frequent small flood events, whereas high-income countries are not significantly affected by relatively small floods, possibly due to flood mitigation measures (e.g., dams, levees, early-warning system, etc.), resulting in a lower mortality rate in higher income countries.

On the other hand, the loss rates showed both negative and positive changes in the past, depending on income level and the target period. This may reflect both a decrease in exposed assets, partly due to improved flood mitigation measures or other flood risk measures, and an increase in exposed assets due to economic growth in flood-prone regions. Even if the frequency and magnitude of flooding and flood mitigation measures are unchanged, exposed assets will increase with economic growth in flood regions. The inverted U-shape trend in upper-middle, lower-middle and low income countries demonstrates this balance: as indicated by several previous studies^19^,^20^, this trend shows that the flood risk first increases because improvements in flood mitigation measures progress more slowly than increases in assets; the risk then decreases with accumulation of wealth (Fig. 3c). As the increasing rate of population, and hence the increasing the modelled exposed population, is smaller than the increasing rate of modelled exposed GDP (Fig. 1b,c), inverted U-shape curves are not clearly seen in mortality rate in lower-middle and upper-middle countries. This can be confirmed by calculating loss rate using fixed GDP at the level of 2000: the inverted U-shape pattern is not clearly seen in lower-middle and upper-middle income countries.

A previous similar work (J15^10^) analysed changes in flood vulnerability between 1990 and 2010, whereas the present study focused on a longer time period, from 1960 to 2013. Although overall changes in global mortality rates and loss rates between J15 and this study showed similar patterns, several differences were observed. For example, the mortality rate in the J15 decreased globally and the improvement in mortality rate was faster in countries with lower income levels. In our analysis of the same period, the global mortality rate did not show a significant decreasing trend, because mortality rates in higher income countries did not show significant changes in recent decades, while those in lower income countries showed a negative trend, as seen in J15 (Supplementary Table S1). On the other hand, global loss rates showed a decreasing trend in both J15 and our study for the period 1990–2010. However, as all countries except for high-income countries show an inverted U-shape pattern of loss rate, we cannot obtain a simple positive or negative trend in loss rates in longer periods.

The differences in mortality rate and loss rate could result from differences in disaster statistics and modelled flooded areas. We utilised the EM-DAT for flood disaster statistics, which adopts a ranking method to select sources of disasters according to data from the United Nations, governmental and non-governmental agencies, insurance companies, research institutes and press agencies^6^. In contrast, J15 utilised Munich Re data, which includes additional information, such as national insurance agency reports, as well as reports from clients and loss adjusters, insurance-related journals, world weather services and scientific institutes^25^. This difference in the source of flood disaster statistics, as well as the order of priority of the sources, may be the cause of the discrepancy in reported flood damage values. Loss rates in our study were 2–10 times lower than those in J15 in the period 1990–2010. The large discrepancies in loss rates between J15 and this study may be a result of the GDP data used to calculate modelled exposed GDP. The calculation in J15 used GDP per capita at purchasing power parity (PPP) values, with base-year 2010 as the modelled exposed GDP value, but our study used the nominal value of GDP to calculate modelled exposed GDP, because extrapolations of the GDP deflator and PPP conversion factor have large uncertainties, especially for the period before 1990.

Despite the similarity between J15 and this study, in terms of the model frameworks for calculating flooded areas, differences in spatial resolution – in particular for topographic data, population distribution maps or the use of climate forcing to drive models – could have led to the differences in modelled flood exposure. A recent global flood model intercomparison study showed that the global river and inundation model used in J15 and our study showed similar inundation distribution over Africa^32^. A sensitivity test of modelled flood exposure using high-resolution population data (GRUMPv1^33^) showed that although differences in calculated flood exposure due to different horizontal resolution or different source of population data occur, the annual variation and hence long-term trend of mortality rates are similar. We therefore think that the largest contribution to the difference in loss rates is from the difference in disaster statistics used in the two studies.

These similarities and differences in flood disaster statistics or modelled flooded area provide independent information on past changes in flood vulnerability, increasing the credibility of the findings of J15. More importantly, the analysis over a longer time period in this study enabled statistical analysis of the significance of past trends. Because improvements in flood protection levels are more obvious before the 1990 s in many developed countries, and because economic growth in many countries is more clearly seen in past records, analysis of flood vulnerability over a longer time period added information about the effect of the balance between increases in assets or population in flood-prone regions and improvements in flood mitigation measures, as shown by the inverted U-shape obtained for all income levels except the high-income level.

We calculated flood exposure based on modelled annual maximum daily flood water depth. This means flood exposure may be underestimated if more than one flood occurs in a single year. The effects of flood damage due to long inundation periods, for example because of industrial activity stoppage, were not included in our modelled exposed GDP calculations. Because the number of reported flood events in the EM-DAT was small, in particular for low-income countries before the 1990 s, calculated flood vulnerabilities in earlier periods were biased to reflect this limited number of flood events. However, it was assumed that historical disastrous floods causing relatively severe damage were recorded in the EM-DAT in this period, as the modelled flood exposure can reflect the past trend of reported flood damage (Supplementary Figs S4 and S5). Hence, the calculated global or regional average flood vulnerability could be biased to reflect the flood vulnerability in well-reported countries, which can underestimate vulnerability, or in countries that have had relatively severe flooding in the past.

The historical change of flood vulnerability is complex and dynamic, depending on economic, social, demographic, cultural, institutional, and governance factors^2^. For example, Mozambique displaced more than 465,000 people in 2001, 2007 and 2008 to reduce flood risk, and more than 1,000,000 people were displaced in the USA after Hurricane Katrina^34^. Such local-scale movements of people cannot be fully resolved in the global-scale population map. The adaptation effect (the experience of a flood event significantly reduces the impacts of flood events that occur shortly after it) was found to have reduced flood vulnerability in Prague (1997 and 2002), along the Meuse River (1993, 1995), in Bangladesh (1971, 2007) and Mozambique (2000 and 2007) and along the Parana River (1983 and 1992)^35^. On the other hand, the reduction of small but frequent floods caused by flood control infrastructures could increase flood risk due to reduced awareness of flooding, a phenomenon known as the levee effect^36^. These non-linear and discontinuous effects could have contributed to past changes in flood vulnerability.

## Conclusion

In this study, long-term trends in flood vulnerability between 1960 and 2013 were analysed. In accordance with a previous study, global mortality rates and global loss rates showed a decreasing trend (Fig. 2), and inverse relationships were found between flood vulnerability and GDP per capita (Fig. 3), indicating an improvement in flood vulnerability at the global scale since 1960 associated with economic growth. However, although a significant negative trend in global mortality rate was seen across the whole analysis period (1960–2013), the rate for the most recent period (1990–2010) did not show a statistically significant trend (Supplementary Table S1). This result highlights the importance of the analysis period, as well as uncertainties, when calculating flood vulnerability. The long-term trend in loss rate varies between different income levels, due to the balance between improvements in flood mitigation measures and increases in assets in flood-prone regions associated with economic growth (in particular in developing countries).

A comparison of the mortality rate calculated from the real population and a population of fixed distribution since 1960 showed that more than 24 countries out of the 113 analysed showed a large difference (>20%) in mortality rate; however, the increase or decrease in mortality rate varies across regions. This means that the effect of changes in population distribution on flood vulnerability is not so simple, and hence future flood exposure may be under- or overestimated when only future projected population data is used without considering spatial changes in population density.

## Methods

### Modelled flood exposure calculation

Modelled flood exposure (the number of people or the value of assets potentially affected by flooding) at 5′ × 5′ (approximately 10 km × 10 km at the equator) was calculated using a global river and inundation model, the Catchment-based Macro-scale Floodplain model (CaMa-Flood)^26^ from 1960 to 2013. Daily runoff input to CaMa-Flood was obtained from a land surface model, the Minimal Advanced Treatments of Surface Integration and Runoff model, which includes a representation of water table dynamics (MATSIRO-GW)^37^,^38^ at 1.0° × 1.0° resolution, forced by 3-hourly meteorological forcing data based on the Japanese 55-year Reanalysis (JRA55) corrected with observed climate variables (Iizumi *et al*., prep). The CaMa-Flood annual maximum flood water depth at 15′ × 15′ was downscaled to 5′ × 5′ following a method similar to the one used in a previous study^39^ using a high-resolution digital elevation model (DEM)^40^; flooded area fraction (inundation area percentage in a grid cell) at 5′ × 5′ was also calculated. CaMa-Flood reasonably represented the variation and peaks of river discharge (Supplementary Information; Supplementary Figs S1 and S2). The modelled flooded area fraction from CaMa-Flood showed a similar distribution to that derived from satellite observations in 2007 in Bangladesh (Supplementary Fig. S3).

We then counted the number of people and the value of assets affected by flooding by overlaying the modelled flooded area fraction with global gridded population and GDP data. Population data for 1960–2013 was created by distributing the by-country population data of the World Bank database (http://data.worldbank.org/about/country-and-lending-groups) using a 5′ × 5′ population distribution map from the History Database of the Global Environment (HYDE; ver. 3.1)^27^,^28^. HYDE data in 1960, 1970, 1980, 1990, 2000 and 2005 were linearly interpolated to create annual population distributions between 1960 and 2005. The 2005 population distribution was applied to years between 2006 and 2013. Since the land area in CaMa-Flood and the resulting calculated flooded area fraction were smaller than that using HYDE, the population map was adjusted to the land mask of CaMa-Flood by uniformly distributing the population of the grid cell defined as sea in CaMa-Flood to the country nearest the grid cell. A gridded map of GDP was calculated as the population multiplied by the nominal GDP per capita of the World Bank database, assuming that GDP per capita does not change within a country. Following a previous study, we assumed that local people can adapt to small and frequent flooding that occurs almost every year and did not account for flooding with a return period shorter than 2 years in the calculation of exposure^17^.

### Flood vulnerability calculation

Flood vulnerability is defined according to the mortality rate (the ratio of the reported fatalities to the modelled exposed population) and loss rate (the ratio of the reported economic losses to the modelled exposed GDP) following the concept described by Jongman *et al*.^10^ (J15). We calculated mortality and loss rates as the ratios of reported flood loss and damage in the EM-DAT to modelled flood exposures using an equation proposed in J15:


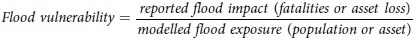


Reported fatalities and economic losses were obtained from the EM-DAT, provided by the Centre for Research on the Epidemiology of Disasters (CRED)^6^. The reported economic loss was given as the nominal value. The sources of reported flood loss and damage in the EM-DAT are informed by data from the United Nations, governmental and non-governmental agencies, insurance companies, research institutes and press agencies. Flood events were recorded in the EM-DAT if at least one of the following criteria was fulfilled: 10 or more people dead, 100 or more people affected, or the country declared an emergency or required international assistance. However, many events that meet the criteria are often not recorded in the EM-DAT. It is also noted that because we selected flood events recorded in the category of “hydrological group” within the category of “natural disasters”, we cannot separate fluvial from coastal flooding. In total, 4,158 flood events for the period 1960–2013 were used in this study. As the EM-DAT provides information on reported fatalities and economic losses at the country level, we calculated the flood vulnerability of each country over the period 1960–2013. The loss rate was calculated from modelled exposed GDP using the nominal GDP per capita and nominal economic loss from the EM-DAT. We could not calculate mortality or loss rates in Albania, the Bahamas, Barbados, Hong Kong, Grenada, Saint Lucia, Trinidad and Tobago, the Soviet Union, Yugoslavia, Saint Kitts and Nevis, Libyan Arab Jamah, Taiwan and North Korea due to data availability and reproducibility of the global river and inundation model (Supplementary Table S2).

The calculated flood vulnerabilities were analysed across four country income levels (low, lower-middle, upper-middle and high) and six regions (East Asia, Europe and Central Asia, Latin America and the Caribbean, North America, South Asia, and Sub-Saharan Africa) classified by the World Bank (base year = 2014).

### Effect of population distribution change on flood vulnerability

To analyse the effect of past changes in population distribution within a country on flood vulnerability, we compared the modelled exposed population using the real population map (RPOP) with that calculated using a population map without consideration of historical changes in population distribution within a country by fixing the population distribution to that observed in 1960 but scaling the population to the RPOP (FPOP). As RPOP is based on the HYDE 3.1 dataset^27^,^28^, it included estimations of underlying demography related to urban built-up areas and land use changes (cropland and grassland); this allowed us to capture population shifts from rural to urban regions at a country scale. Because the FPOP country population was adjusted to be identical to that of RPOP, we could not capture the effect of population shifts to other countries on modelled flood exposure and flood vulnerability. We assessed the effect of changes in population distribution on mortality rate (A) using the mortality rates calculated from RPOP (RVUL) and FPOP (FVUL):


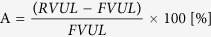


## Additional Information

**How to cite this article**: Tanoue, M. *et al*. Global-scale river flood vulnerability in the last 50 years. *Sci. Rep.* **6**, 36021; doi: 10.1038/srep36021 (2016).

**Publisher’s note:** Springer Nature remains neutral with regard to jurisdictional claims in published maps and institutional affiliations.

## Supplementary Material

Supplementary Information
