# Supplementary material for: Global-scale river flood vulnerability in the last 50 years
Source: Sci Rep. 2016 Oct 26;6:36021. doi: 10.1038/srep36021 (PMC5080543; doi:10.1038/srep36021)
Supplement: Supplementary Information [file srep36021-s1.pdf]

## Supplementary information

# Global-scale river flood vulnerability in the last 50 years

Masahiro Tanoue, Yukiko Hirabayashi and Hiroaki Ikeuchi

**Validation of modelled discharge and flooded area fraction**

Modelled discharges using CaMa-Flood were compared with observed daily discharges at 87 river basins, obtained from the Global Runoff Data Centre (GRDC; http://www.bafg.de/GRDC/EN/Home/homepage_node.html). We selected 87 gauging stations with an upstream area larger than 150,000 km$^2$ and with at least 20 years of available data (within the period 1960–2010) for the analysis. The number of gauging stations with a statistically significant correlation (95% confidence level) between modelled and observed annual mean discharge was 73 out of 87 stations. The number of stations with a statistically significant correlation for annual maximum discharge was 49. The correlation coefficients between modelled and observed annual mean, annual maximum and extreme discharges (corresponding to the 100-year return period) were 0.90, 0.83 and 0.73, respectively (Supplementary Fig. S1). In addition, the correlations between modelled and observed specific discharge (i.e., river discharge divided by upstream area) were also high (0.86 for annual mean, 0.80 for annual maximum and 0.68 for extreme discharge) (Supplementary Fig. S2). The annual mean, annual maximum and extreme river discharges were within a bias of < 50% for 54, 50 and 42 of 87 stations, so these results were within an acceptable range for global analysis.

Potential biases in the retrospective inundation simulations arose from biases in the climate forcing parameters and uncertainty in land surface processes, including snow melt and evapotranspiration. Artificial river flow regulation, such as reservoir operation and water intake, could also lead to inconsistencies, because CaMa-Flood does not consider these effects.

Besides river discharge, a modelled flooded area fraction was compared with satellite observation. Supplementary Fig. S3 (a) and (b) shows an example of annual maximum modelled flooded area fraction (inundation area percentage of a grid cell) and observed maximum flooded area fraction derived from Moderate Resolution Imaging Spectroradiometer

(MODIS) data[41] over the north-eastern part of Bangladesh (21°–27° N, 86°–95° E) in 2007. CaMa-Flood could capture the spatial pattern of the large flooded area fraction (> 0.5), but underestimated flooding along the main river stream in the central region and along the coastal region of Bangladesh. One reason for the underestimation along the main river stream in the central region is the underestimation of river channel depth, which was empirically estimated in the model[39]. One reason for the discrepancy in flooded area fraction along the coastline is the mismatch of the river routing maps of CaMa-Flood and land-sea mask, especially in low deltaic regions. The effect of the storm surge tide due to cyclone Sidr, which was not implemented in the model, may also have led to the underestimation and the discrepancy.

**Comparison of modelled flood exposure and reported flood loss and damage**

The modelled exposed population was compared to observed reported fatalities at a national scale. Bangladesh was selected because that country has suffered several floods over the past decades. Supplementary Fig. S4 shows the annual variation in reported fatalities and the modelled exposed population in Bangladesh for the period from 1980 to 2013. Reports involving more than 1,000 fatalities were recorded in 1984, 1987, 1988, 1998 and 2007. the modelled exposed population captured these large peaks in the reported fatalities ($R = 0.55$).

The comparison between the modelled exposed population and reported fatalities was then applied globally. We selected 30 countries with reported fatalities and economic losses from flooding data within the EM-DAT data for at least 20 years (within the period 1980–2013) and obtained positive correlations between the reported fatalities and the modelled exposed population for 25 countries (approximately 83.3%) and between the reported economic losses and the modelled exposed GDP for 20 countries (approximately 66.7%) (Supplementary Fig. S5). The results suggest that the modelled exposed population and GDP reflect the variation in magnitude of historical flood loss and damage well.

**References**

42. Kotera, A., Nagano, T., Hanittinan, P. & Koontanakulvong, S. Assessing the degree of flood damage to rice crops in the Chao Phraya delta, Thailand, using MODIS satellite imaging. *Paddy Water Environ.* **14**, 271–280 (2016).

**Supplementary Tables**

Table S1. Summary of long-term trends in mortality rate and loss rate for the periods of 1960–2013 and 1990–2010.

| | Mortality rate (1960–2013) | Mortality rate (1990–2010) | Loss rate (1960–2013) | Loss rate (1990–2010) |
|---|---|---|---|---|
| Global | -- | | - | - |
| High | -- | | | |
| Upper-middle | -- | | ++ | - |
| Lower-middle | -- | - | -- | |
| Low | -- | -- | | |

The single and double plus (minus) signs indicate positive (negative) trends at the 95% and 99% significance levels, respectively. The statistical significances of the long-term trends were established by the Mann–Kendall test.

Table S2. Summary of missing flood vulnerability data.

| Country | Missing all mortality rate data | Missing all loss rate data | Reason |
|---|---|---|---|
| Albania, Grenada | Yes | Yes | No flood record in the EM-DAT |
| Bahamas, Barbados, Hong Kong, Saint Lucia, Trinidad and Tobago | Yes | Yes | Not resolved in our global model |
| Soviet Union, Yugoslavia, Saint Kitts and Nevis, Libyan Arab Jamah | Yes | Yes | Not defined in the HYDE country mask data |
| Taiwan, North Korea | No | Yes | Nominal GDP data was unavailable in the World Bank database |

**Supplementary Figures**

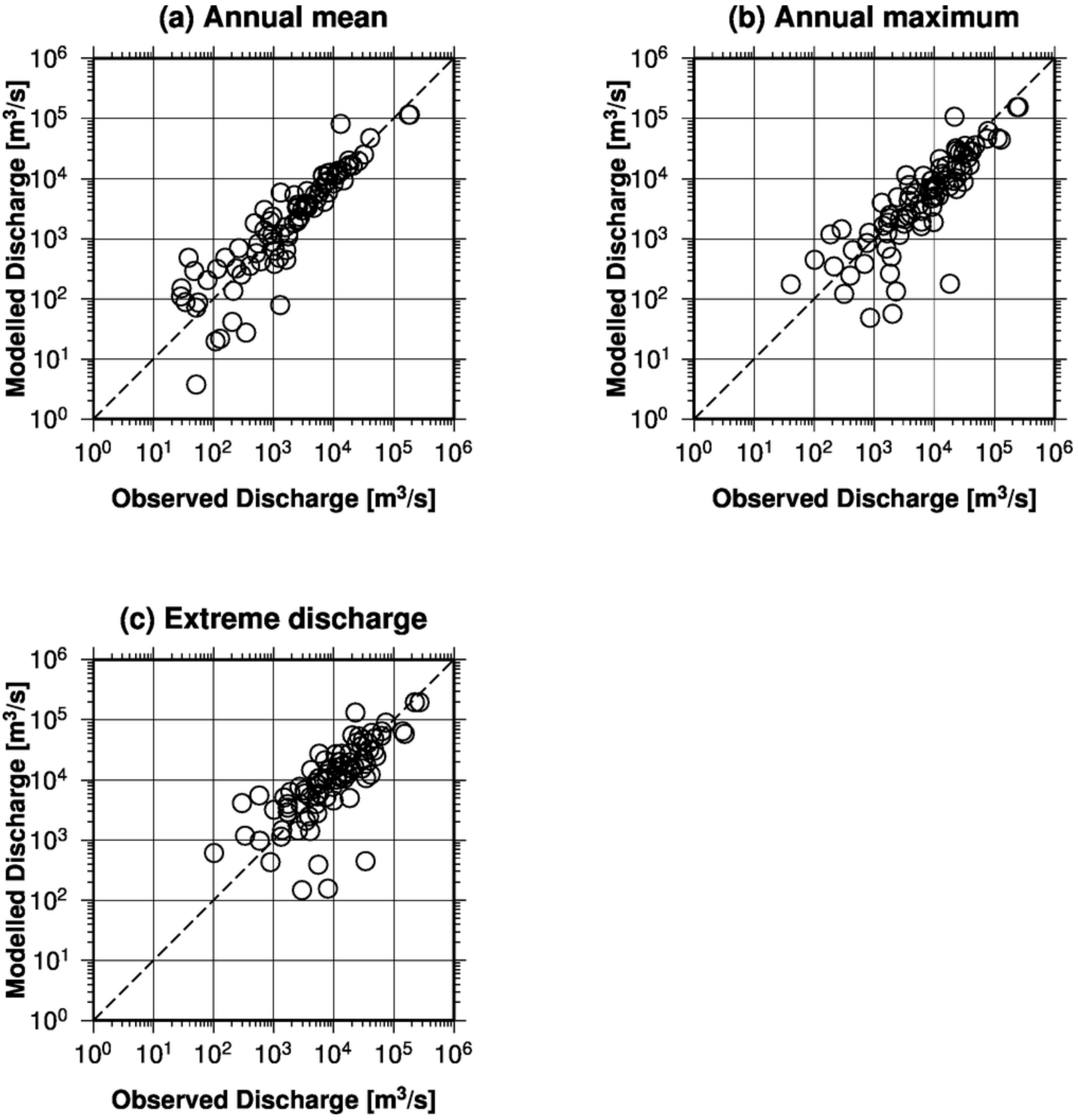


Figure S1. Relationship between modelled and observed discharge for the period from 1960 to 2010 at 87 selected gauging stations: (a) daily mean, (b) annual maximum, and (c) extreme discharge (corresponding to the 100-year return period). This figure was created using Generic Mapping Tools 4.5.6.

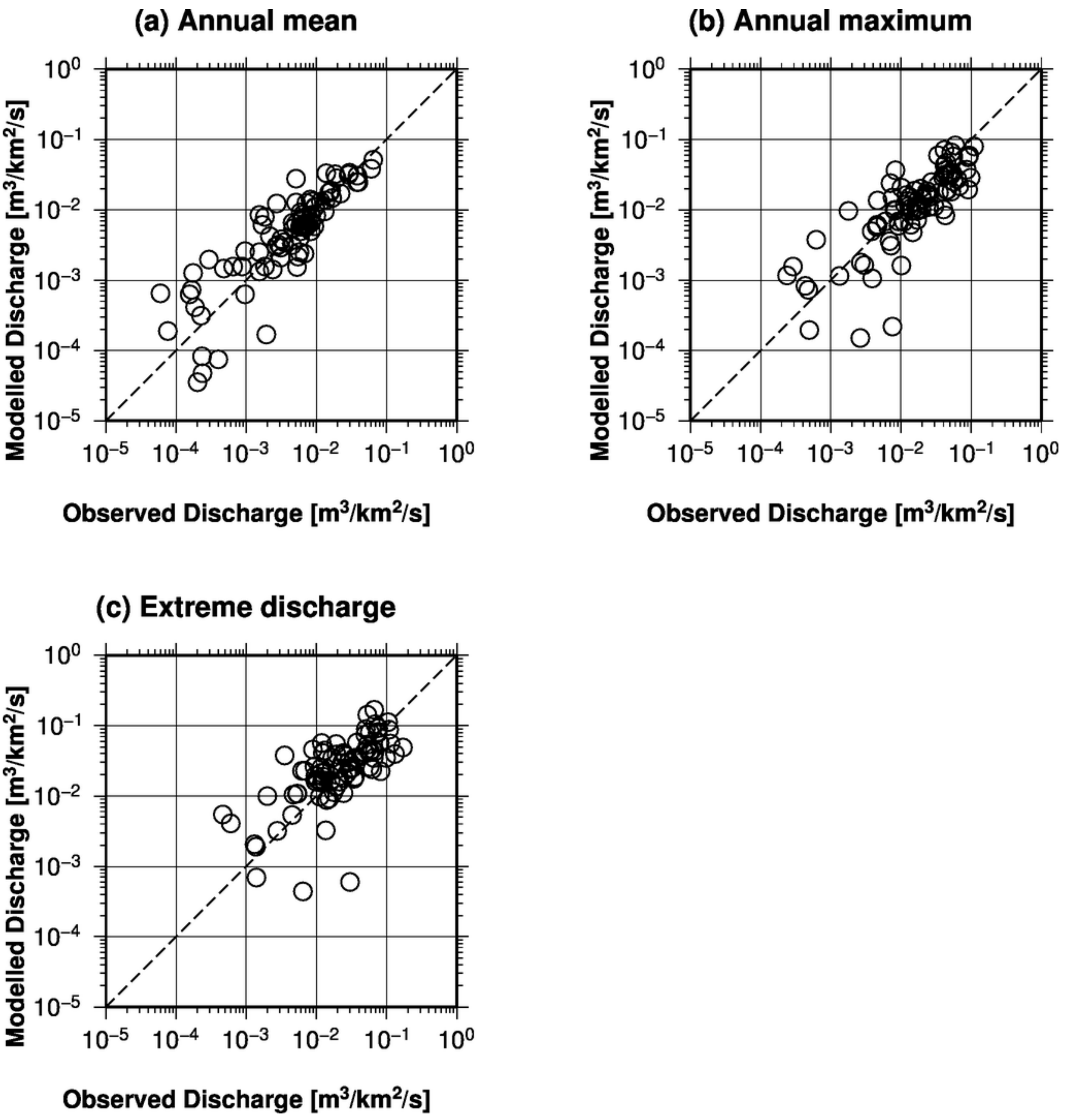


Figure S2. Relationship between modelled and observed specific discharge for the period from 1960 to 2010 at 87 selected gauging stations: (a) daily mean, (b) annual maximum, and (c) extreme discharge (corresponding to the 100-year return period). This figure was created using Generic Mapping Tools 4.5.6.

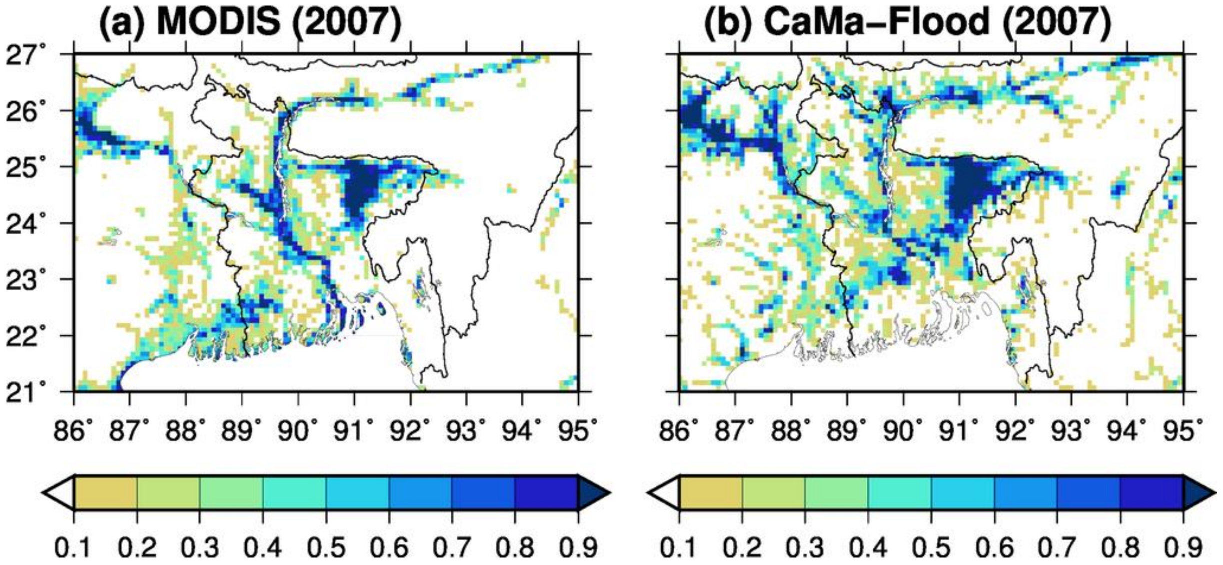


Figure S3. Annual maximum flooded area fraction in 2007 (a) derived from satellite observations[42] (Moderate Resolution Imaging Spectroradiometer; MODIS) and (b) modelled using the Catchment-based Macro-scale Floodplain model (CaMa-Flood). This figure was created using Generic Mapping Tools 4.5.6.

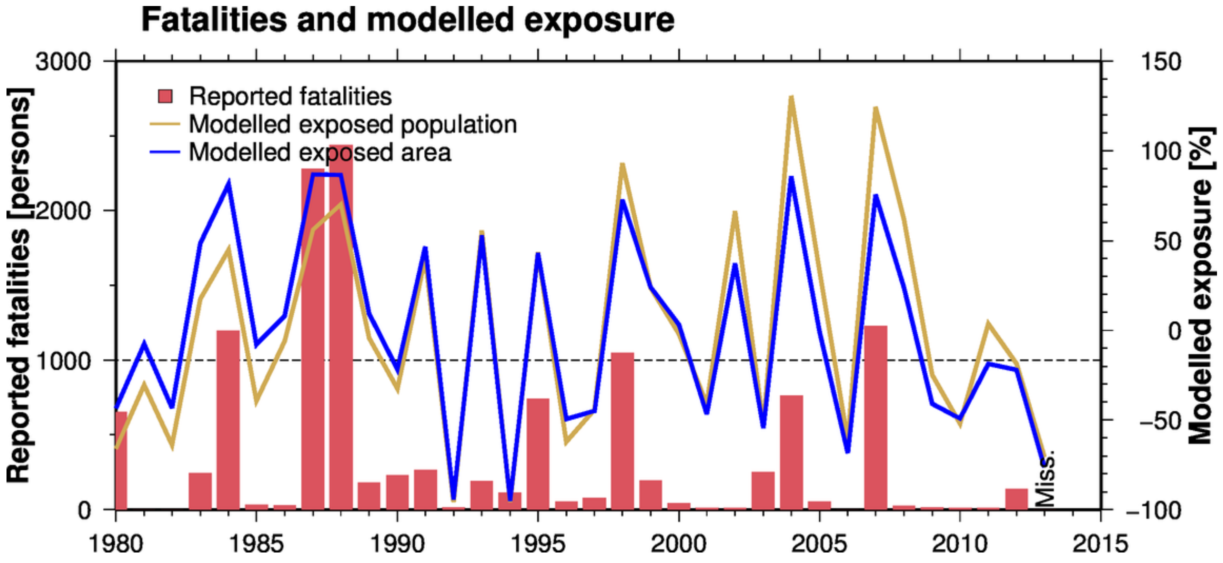


Figure S4. Reported fatalities from flooding in the EM-DAT (bar), annual the modelled exposed population (orange line) and annual flooded area (blue line) in Bangladesh. The modelled exposed population and flooded area are relative changes compared to the average across 1980–2013. This figure was created using Generic Mapping Tools 4.5.6.

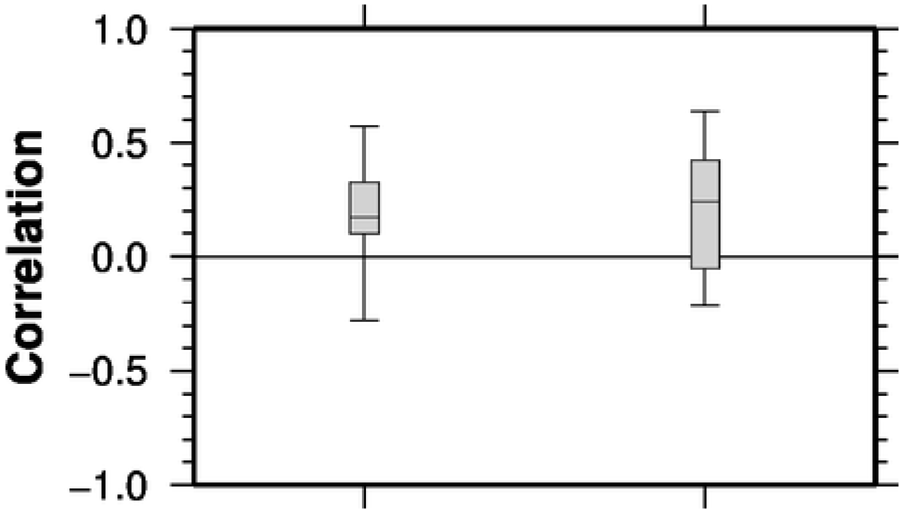


Figure S5. Boxplot of correlation coefficients between the modelled exposed population and reported fatalities (left) and between modelled exposed GDP and reported economic losses (right) for 30 selected countries for the period from 1980 to 2013. The boxes represent the 25th and 50th percentiles, and the whiskers show the minimum and maximum correlations. This figure was created using Generic Mapping Tools 4.5.6.
